# Supplementary material for: Surface Coordination of Pd/ZnIn2S4 toward Enhanced Photocatalytic Activity for Pyridine Denitrification
Source: Molecules. 2022 Dec 29;28(1):282. doi: 10.3390/molecules28010282 (PMC9822349; doi:10.3390/molecules28010282)
Supplement: Supplementary file 1 [file molecules-28-00282-s001.zip › molecules-2069819-supplementary.pdf]

Article

Surface Coordination of Pd/$ZnIn_2S_4$ toward Enhanced Photocatalytic Activity for Pyridine Denitrification

Deling Wang [1], Erda Zhan [1], Shihui Wang [1], Xiyao Liu [2,3], Guiyang Yan [2,3,*], Lu Chen [2,3,*] and Xuxu Wang [1,*]

1 State Key Laboratory of Photocatalysis on Energy and Environment, Fuzhou University, Fuzhou 350002, China
2 Province University Key Laboratory of Green Energy and Environment Catalysis, Ningde Normal University, Ningde 352100, China
3 Fujian Provincial Key Laboratory of Featured Materials in Biochemical Industry, Ningde Normal University, Ningde 352100, China
* Correspondence: ygyfjnu@163.com (G.Y.); chenlu199104@163.com (L.C.); xwang@fzu.edu.cn (X.W.); Tel.: +86-13809566652 (G.Y.); +86-156959097359(L.C.); +86-13600887951(X.W.)

Table S1. Actual mass content of Pd from EDS analysis of the prepared samples.

Sample	Theoretical proportion (%)	Actual proportion (%)
Pd/ $ZnIn_2S_4$	1	0.90

Table S2. Comparison between the photocatalytic activity of 1% Pd/ ZnIn2S4 and that of other reported catalysts for pyridine denitrogenation.

Photocatalysts	C pyri-dine (ug/g)	C cat (mg/mL)	Light Source	Denitrogenation efficiency (%)	Ref.
Pd/$ZnIn_2S_4$	100	1.0	300W(λ ＞420nm)	4.0h,81%	This work
TiO_2@MIL-101(Cr)	100	1.0	300W(λ ＞420nm)	4.0h,70%	[R1]
Bi_2MoO_6/CdS	100	1.0	300W(λ ＞420nm)	4.0h,81%	[R2]
$Bi_{20}TiO_{32}$	100	0.5	400W(λ ＞420nm)	2.5h,86%	[R3]
CoCu-ZIF	100	1.0	300W(λ ＞420nm)	4.0h,80%	[R4]

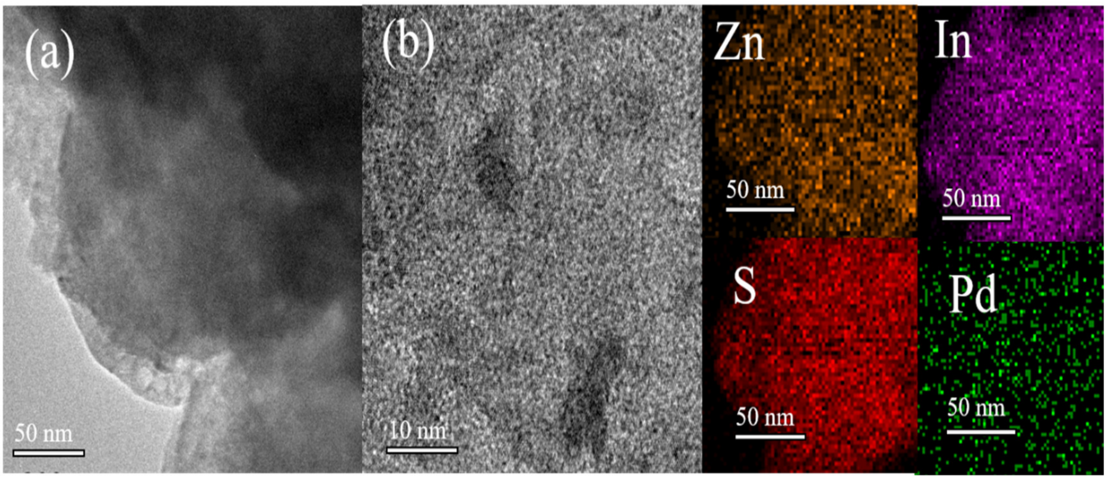

Figure S1. (a, b) HRTEM of 1% Pd/ZIS after 5 times recycle and element mapping images

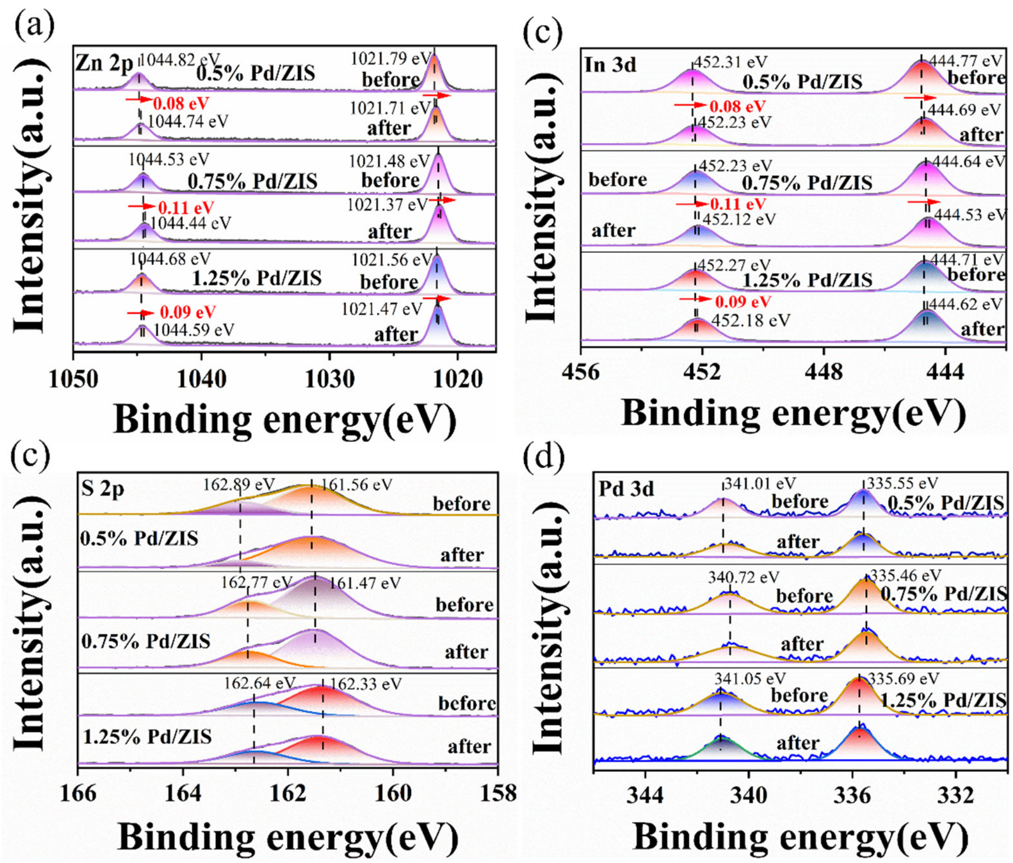

Figure S2. XPS spectra of the other ratio Pd/ZIS sample before and after pyridine adsorption.

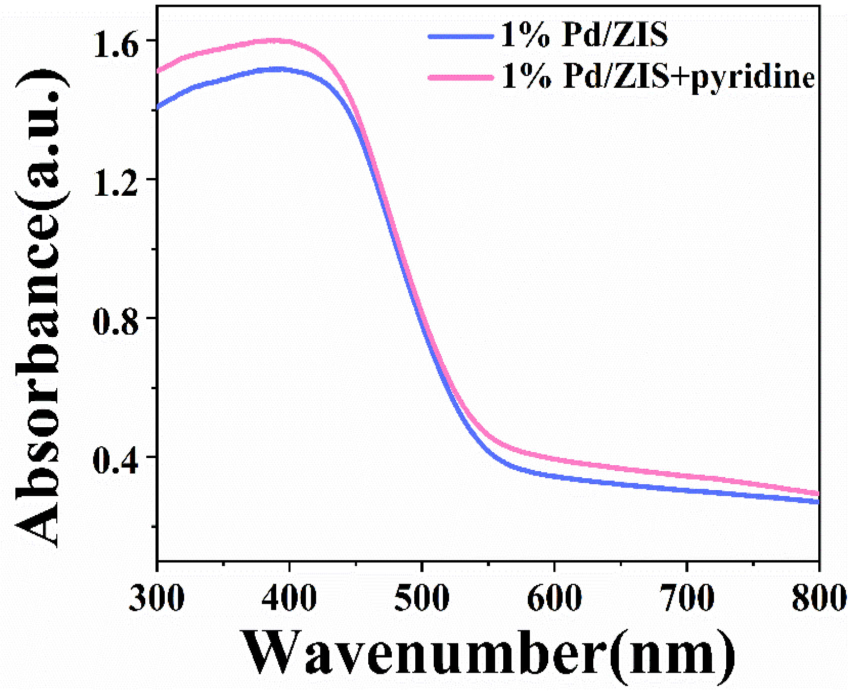

Figure S3. UV -vis DRS spectra of the 1% Pd/ZIS before and after pyridine adsorption.

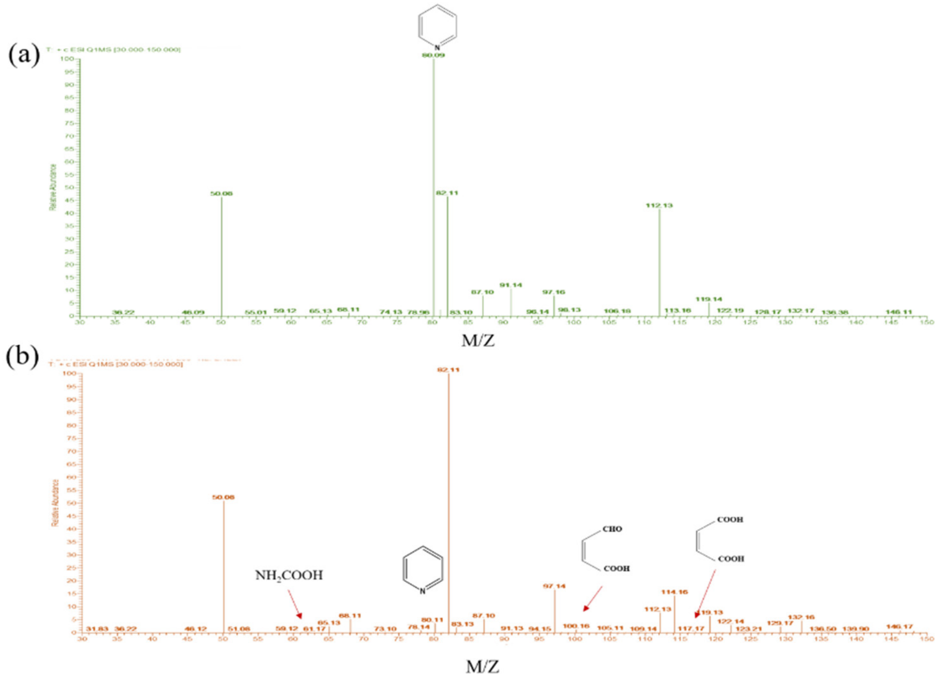

Figure S4. High-performance liquid chromatography profiles of pyridine after different irradiation times: (a) 0 and (b) 4 h.

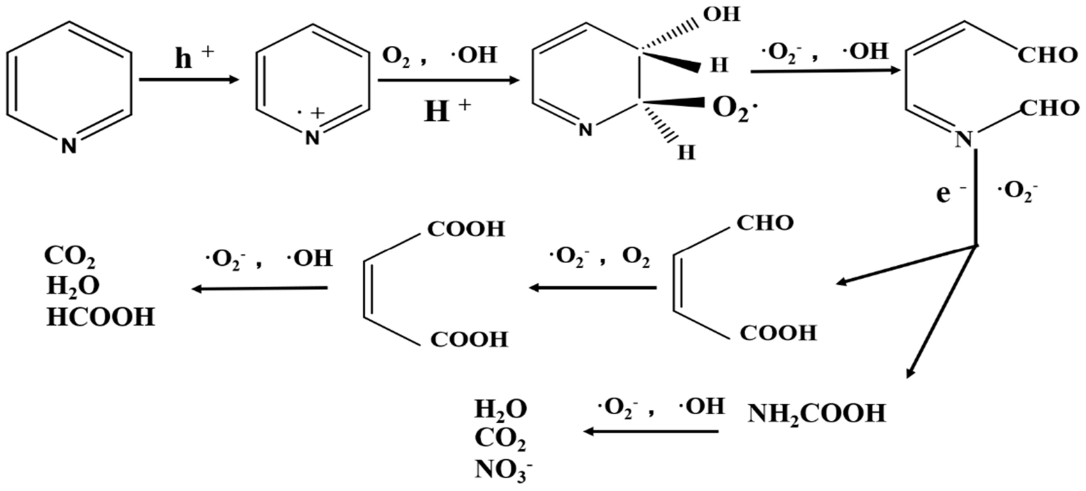

Figure S5. Possible denitrification pathway of pyridine.

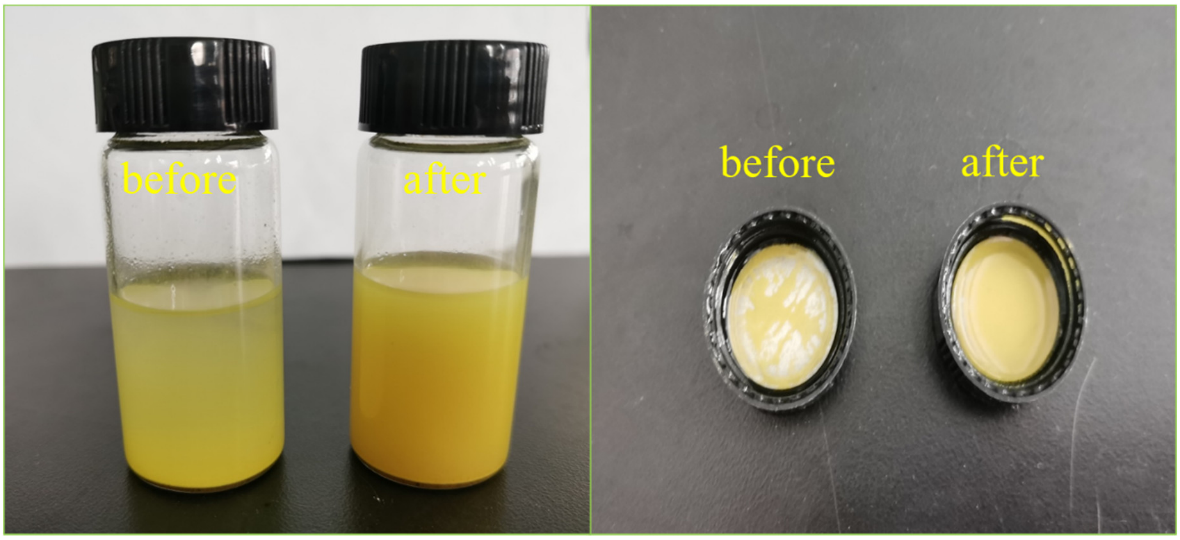

Figure S6. Dispersion experiment of before and after CTAB absorption

As shown in Figure S5, CTAB serves as a "bridge molecule" between the hydrophilic surface of Pd/ZIS and hydrophobic octane molecules. The dispersion experiment indicated that the addition of CTAB improved the dispersibility of Pd/ZIS hybrids in octane.

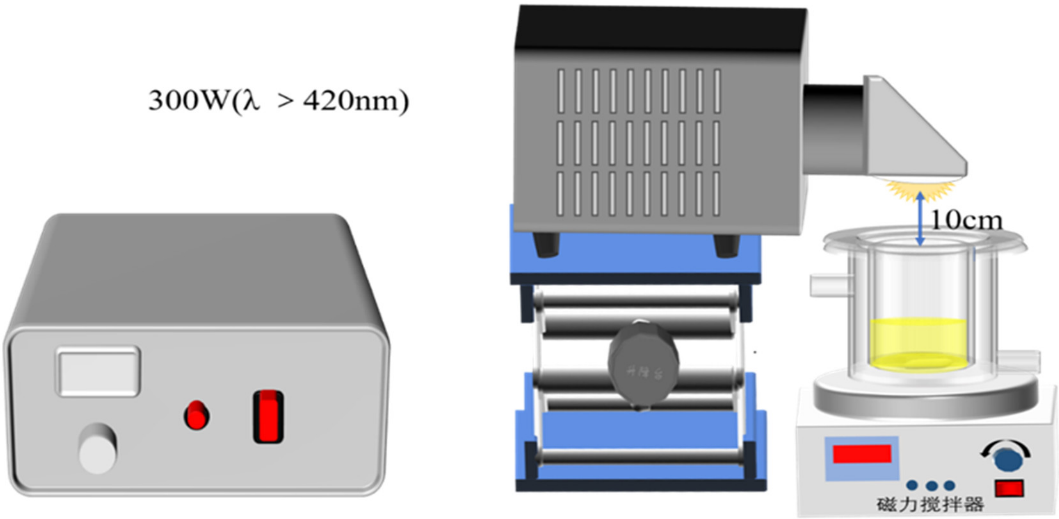

Figure S7. Schematic diagram of photocatalytic fuel denitrification reaction

Table S3. Pyridine peak area from LC-MS analysis of the prepared samples.

Sample	Before adsorption	After dark adsorption(1h)	Difference	Pyridine adsorption value(mg)	After light adsorption(4h)	Difference	Pyridine adsorption value(mg)
$ZnIn_2S_4$	100	98	2	0.001	55	43	0.043
1% $ZnIn_2S_4$	100	95	5	0.005	6	89	0.089

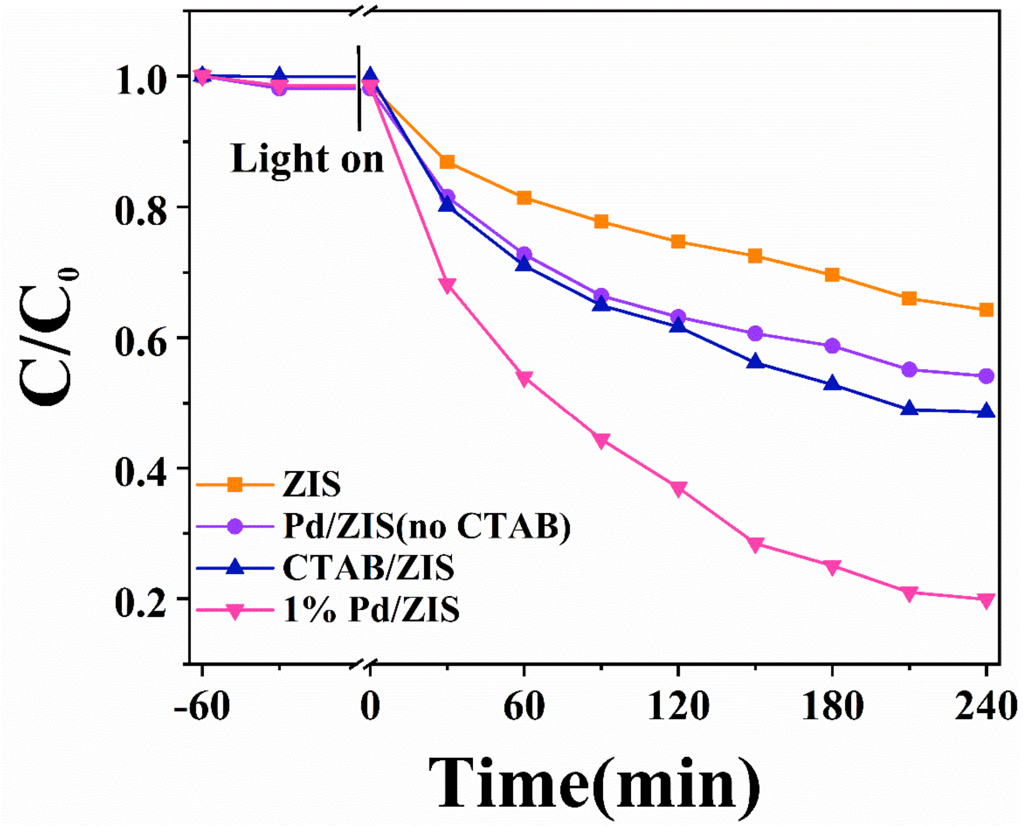

Figure S8. Photocatalytic denitrification of pyridine under visible light irradiation over ZIS, Pd/ZIS (no CTAB), CTAB/ZIS, and 1% Pd/ZIS.

References

[R1] Lu, Y.; Liang, R.; Yan, G.; Liang, Z.; Hu, W.; Xia, Y.; Huang. R. Solvothermal synthesis of TiO_2@MIL-101(Cr) for efficient photocatalytic fuel denitrification. *Journal of Fuel Chemistry and Technology*. 2021, *50*, 1-8. https://kns.cnki.net/kcms/detail/14.1140.tq.20211025.1659.002.html.

[R2] Hu, W.; Jiang, M.; Liang, R.; Huang, R.; Xia, Y.; Liang, Z.; Yan, G. Construction of Bi_2MoO_6/CdS heterostructures with enhanced visible light photocatalytic activity for fuel denitrification. *Dalton Trans*. 2021, *50*, 2596-2605. https://doi.org/10.1039/D0DT03922E.

[R3] Zheng, L.; Yan, G.; Huang, Y.; Wang, X.; Long, J.; Li, L.; Xu. T. Visible-light photocatalytic denitrogenation of nitrogen-containing compound in petroleum by metastable $Bi_{20}TiO_{32}$. *International Journal of Hydrogen Energy*. 2014, *39*, 13401-13407. https://doi.org/10.1016/j.ijhydene.2014.04.027.

[R4] Lu, Y.; Pan, H.; Lai, J.; Xia, Y.; Chen, L.; Liang, R.; Yan, G.; Huang. R. Affiliation Bimetallic CoCu-ZIF material for efficient visible light photocatalytic fuel denitrification. *RSC Advances*. 2022, *12*, 12702-12709. https://doi.org/10.1039/D2RA01049F.
